# Effects of Antiepileptic Drug Tapering on Episodic Memory as Measured by Virtual Reality Tests

**DOI:** 10.3389/fneur.2020.00093

**Published:** 2020-02-20

**Authors:** Yvonne Höller, Christopher Höhn, Fabian Schwimmbeck, Gaën Plancher, Eugen Trinka

**Affiliations:** ^1^Faculty of Psychology, University of Akureyri, Akureyri, Iceland; ^2^Department of Neurology, Christian Doppler Medical Centre and Centre for Cognitive Neuroscience, Paracelsus Medical University, Salzburg, Austria; ^3^Department of Psychology, University of Salzburg, Salzburg, Austria; ^4^Laboratoire EMC, Mémoire, Émotion et Action, Université Lumiére Lyon 2, Lyon, France

**Keywords:** virtual reality, episodic memory, epilepsy, epilepsy monitoring unit, antiepileptic drugs

## Abstract

Antiepileptic drugs impair episodic memory in patients with epilepsy, but this effect has so far only been examined with tests that do not provide first-person experience—an aspect that is crucial for episodic memory. Virtual reality techniques facilitate the development of ecologically valid tests. In the present study, we measure the effect of antiepileptic drug changes in a within-subject design using a virtual reality test in order to provide direct evidence for effects of antiepileptic drugs on episodic memory. Among 106 recruited patients, 97 participated in a virtual reality test up to six times during a 4-day hospitalization, and 78 patients underwent changes in drug load during this period. There were six parallel versions of a virtual town test, with immediate recall and delayed recall after about 12 h. The test requires recall of elements, details, sequence of experience, and egocentric and allocentric spatial memory. We determined drug load by defined daily dose, and compared test performance at lowest antiepileptic drug load to highest antiepileptic drug load. Across the six towns, performance was lower in delayed compared to immediate recall. There was an overall effect of medication when comparing patients taking vs. not taking antiepileptic drugs and/or psychoactive drugs (*p* = 0.005). Furthermore, there was a within-subject effect of antiepileptic drug load (*p* = 0.01), indicating lower test performance at higher drug load. There was no effect of gender, daytime, circadian type, depression, seizures, lesions, and epilepsy. For patients with temporal lobe epilepsy, there was no effect of lateralization. The present study provides direct evidence for episodic memory impairment due to antiepileptic drugs, suggesting that a small change in drug load can matter. This study can serve as a proof of principle for the methodology, but a larger sample is needed to examine the differential effects of individual antiepileptic drugs.

## 1. Introduction

Memory complaints cause severe impairment of quality of life in patients with epilepsy (1–3). Antiepileptic drugs have effects on memory, but the nature and extent of the effect depends on the type of drug and on the domain of memory (4–9). While the effect of antiepileptic drugs on semantic memory and executive functions has been very well examined (10, 11), only a couple of studies have examined the effect of antiepileptic medication on episodic memory. The examination of drug effects on episodic memory deserves more attention. The main motivation for increasing scientific efforts in the documentation of drug effects is the demand from patients for a well-balanced tradeoff between optimal seizure control and minimal side effects. In addition, pharmacological treatment is a source of confound when comparing healthy controls to patients, because patient groups are usually on pharmacologial treatment. However, only recently have scientists developed adequate means for examining episodic memory.

Measuring episodic memory problems has moved to the focus of scientific endeavors, because standard neuropsychological tests are poorly correlated with subjective memory complaints (12, 13). Virtual reality tests realize self-motion for the construction of spatial memory representations. Therefore, these tests allow episodic memory to be assessed in a way that is closer to the original definition of episodic memory (14–16). For spatial episodic memory content, the formation of memories is said to be experience-dependent (17). Virtual reality tests require active engagement and induce the experience of first-person movement through a virtual environment. Thus, they promote egocentric information storage (18). Tests relying on virtual reality are able to determine cognitive functions in pathological aging, which are subjectively relevant and closely related to the memory requirements of daily life (19). Episodic memory induced by virtual environments can be assessed in sub-domains, such as the memory for elements, details describing these elements, time information, and spatial representations of these elements (19). The ecological validity of virtual reality measures was demonstrated through correlation with subjective estimations of cognitive abilities in patients with epilepsy (20).

Virtual reality environments were used in the past to examine spatial navigation (18, 20–22). Spatial memory assessed by virtual environment tasks includes allocentric and egocentric memory (19, 23). These two subscales describe different aspects of episodic memory. Allocentric spatial memory represents stored information about the configuration of elements relative to each other, while egocentric spatial memory includes the first-person perspective of the external world and the representation of the individual in relation to external-world elements. In a series of studies, a virtual reality version of the holeboard was used to examine spatial learning and memory in patients with refractory temporal lobe epilepsy (21, 24). Both studies found a difference in performance between patients and controls, which was ascribed to temporal damage. However, these patients with refractory epilepsy were all treated by medication.

A few studies have examined the effect of antiepileptic drugs in a within-subject design. Ciesielski et al. (25) reported that add-on pregabalin but not the add-on levetiracetam impairs episodic memory in a story recall test. Kamboj and Curran (26) found negative effects of lorazepam and scopolamine on anterograde recognition memory in a similar task using narrative content. Jóźwiak et al. (27) reported negative effects of adjunctive eslicarbazepine on episodic memory in children based on word or picture recognition. However, these studies on the effects of antiepileptic drugs on episodic memory did not test episodic memory in its narrow definition of experience-dependent memory formation (17).

So far, studies comparing episodic memory between patients with and without epilepsy are not able to disentangle the effect of medication from the effect of epilepsy, as patients with epilepsy are always treated with antiepileptic drugs. Other studies compared antiepileptic drug effects within subject but did not use ecologically valid tests for episodic memory. An exception is the work by Lah et al. (28). The authors reported a negative association between the number of antiepileptic drugs and items recalled on a test of autobiographical memories in patients with temporal lobe epilepsy. However, as a cross-sectional study, it is not easy to differentiate the short-term effects of medication from the long-term effects of medication in interaction with severity of epilepsy. Therefore, within-subject designs, comparing cognitive function in one patient at different levels of drug load, are highly warranted. In this study, we take advantage of the setting of the epilepsy monitoring unit, where drug tapering and up-titration is done routinely for clinical purposes. We assessed episodic memory through the use of six parallel versions of a virtual reality test for episodic memory (29), twice a day. We compared examinations with minimum and maximum drug load within patients in order to answer the question of whether antiepileptic drugs affect episodic memory.

## 2. Materials and Methods

### 2.1. Setting

The current study was conducted during the routine care of the epilepsy monitoring unit of the Department of Neurology, Christian Doppler Medical Center, Salzburg, Austria. Admitted patients undergo the usual diagnostic evaluations, consisting of long-term video-EEG monitoring. Recordings are performed over a maximum period of 5 days (Monday to Friday). In order to provoke a timely occurrence of seizures during the monitoring period, it is common practice to taper the dosage of antiepileptic drugs and expose patients to sleep deprivation. Informed consent for serious adverse events was completed routinely upon admission.

### 2.2. Ethical Aspects

The study was designed and conducted in accordance with the World Medical Association Declaration of Helsinki and Good Clinical Practice Guidelines. Prior ethical approval was obtained from the ethical committee of Salzburg (415- E/1755/24-2018). Written informed consent was obtained from all participants.

### 2.3. Recruitment of Patients

A consecutive sample of 106 patients was enrolled. All patients who were 18 years and older, able to give written informed consent, and admitted to the epilepsy monitoring unit between 2nd February 2016 and 11th June 2018 were considered for recruitment. Patients are admitted to the epilepsy monitoring unit in order to classify the epilepsy syndrome, for differential diagnosis of suspicious events, for the assessment of seizure frequency, for optimization of medication, or for presurgical evaluation. Each week, at most four patients were admitted to the epilepsy monitoring unit, and at most one patient was enrolled into the study. We strived to recruit patients with an already-ascertained diagnosis of epilepsy or who fitted into the control group well because a diagnosis of epilepsy was unlikely. Based on these criteria, we approached the best-suited patient first.

Participants who were released from the epilepsy monitoring unit with the diagnosis “no epilepsy” were included in the control group. These patients were admitted to the epilepsy monitoring unit due to a single event of unclear nature. Participants in the control group did not have seizures or interictal epileptiform events during their stay in the monitoring unit. The control group data is presented in Höller et al. (30).

Neurological examination also included, in most cases, structural magnetic resonance imaging (MRI) and also, in some patients with intractable epilepsy, single-photon emission computed tomography, positron emission tomography, and neuropsychological examination.

### 2.4. Procedure

**Figure 1:**
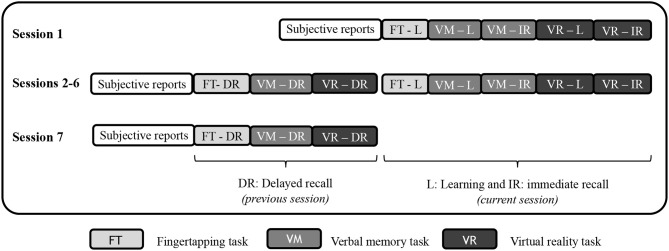
Outline of the procedure during the seven test sessions.

Patients recruited for participation were first tested for chronotype and depression with the German versions of the morningness eveningness questionnaire (31) and Beck's Depression Inventory (32). The participants were then tested at bedside with a Lenovo Laptop (17 inch) on seven occasions for 4 consecutive days, usually from Monday evening until Thursday evening. Test sessions took place in the evening between 6 and 8 p.m. and in the morning between 7 and 9 a.m. The test sessions included a questionnaire about subjective feelings of stress and tiredness, a fingertapping task, a verbal memory task, and the virtual reality test, where only the latter is evaluated and presented here. In the first session, for all three tasks, the learning part was conducted. The verbal memory task and the virtual reality task were followed by immediate recall. The next session started with delayed recall for all three tasks, and then a second version of all three tasks was presented for learning, as well as immediate recall for the verbal memory task and the virtual reality task. Figure 1 illustrates the procedure. The procedure and the order of the six parallel versions were the same for all participants.

### 2.5. Virtual Reality Test

In the *learning part* of the virtual reality test, participants navigated through a virtual town. The task and its six versions are conceptually an update and an extension to six parallel versions of the virtual reality test presented by Plancher et al. (19) and are freely available on Mendeley. (29).

**Figure 2:**
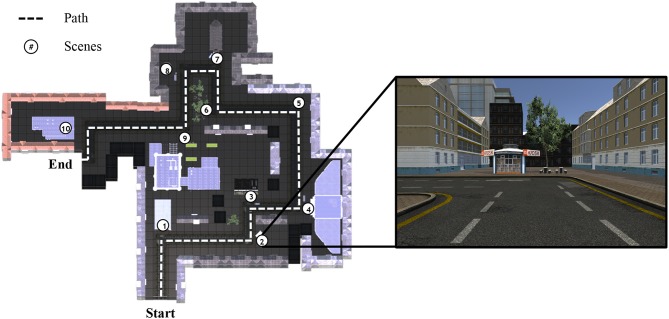
Path from town 1, showing the 10 scenes and the path the participant had to navigate through.

To create six parallel towns, we used UNITY (Unity Technologies ApS, Unity3d.com), including several packages (asset store products) in order to fill the towns with details, such as the super low poly dudes character packs one and two, contemporary city, supermarket, European city buildings, and a power station. We sorted the available details in order to fill 10 scenes per town [nine scenes in the work of Plancher et al. (19)]. In all towns, participants could follow only the main street; there were no options to choose a turn or to get lost. Each scene was at a turning in the street and included at least two elements. An exemplar path (from town 1) is shown in Figure 2. In the second scene, participants could remember a kiosk (element 1), and, to the right of it (allocentric information), two tables with benches (element 2). Behind the benches (allocentric information), there is a tree (element 3). The kiosk is a small house (detail), and there are two benches for each of the two tables (details). After this scene, participants turned left (egocentric information).

**Figure 3:**
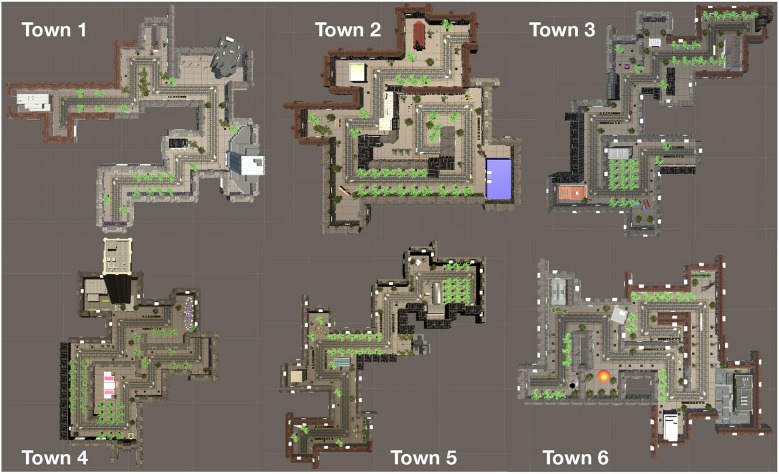
Outline of the six towns.

The total number of elements was balanced across the 10 scenes and six towns. The outline of the six towns is shown in Figure 3. The environment was explored in pedestrian mode; participants used the cursor control keys to navigate.

We also created an empty town of rectangular shape that allowed the participant to go in circles. This town was used on the first day of examination, prior to beginning with the learning part of the first town, in order to familiarize participants with the environment and navigation. In order not to confuse participants with details, there were no details in this training town. Thus, it consisted only of the street and houses to the left and right of the street. The participants moved in the training town until they felt comfortable with the virtual reality environment and navigation, traveling at least one loop through the town.

In the *learning part* of the virtual reality task, participants were instructed to move forward, not to stop, and not to walk too fast. The time spent in the town was recorded automatically by UNITY for later analysis. We informed participants a priori about the questions we would ask in the recall part.

The *recall part* of this test was performed immediately after exploring the town and again about 12 h later. Recall requires the participants to spell out remembered details, which were noted on a structured grid of responses. The participants were asked:

- WHAT: *What did you see in the virtual town?* Patients reported elements that they remembered, such as, e.g., a kiosk, benches, a hospital, a gas station, a traffic light, etc.
- DETAILS: *Can you describe the* <*element*> *in more detail?* For each element that was noted, patients were asked which details they remembered that described the element better.
- WHEN: *When did you see the* <*element*>*: at the beginning, in the middle, or at the end of the town?* For each element they reported, patients were asked when the element had occurred. They were encouraged to judge whether it occurred in the beginning, middle, or toward the end of the walk through the town.
- ALLOCENTRIC WHERE: *Was there anything close to the* <*element*>*, and if so, was it to the left or right, in front of or behind the* <*element*>*?* Patients were asked about the arrangement of the elements relative to each other if they occurred together in a scene.
- EGOCENTRIC WHERE: *After the* <*element*>*, did you turn left or right?* Patients were asked to remember if they turned left or right after the element.

For each of the dimensions WHAT, DETAILS, WHEN, ALLOCENTRIC WHERE, and EGOCENTRIC WHERE, the numbers of correctly reported items were evaluated, rated, and counted from the structured grids of responses. At the end of the study, the authors YH and CH evaluated all responses and preliminary evaluations in order to agree on one evaluation strategy that allowed a consistent appraisal across all patient's responses. For example, number of details turned out to be inconsistent across several raters, such that it was necessary to define a set of valid details. The consensus list of valid items was applied to all patient's responses by one rater (co-author CH) in order to avoid inter-rater bias.

### 2.6. Drug Load

It has been shown that low-dose combinations of multiple drugs are better tolerated than a single high dose, such that it is recommendable to calculate drug load (11, 33, 34). We determined drug load for each day as follows. For each antiepileptic or psychoactive drug, we calculated the ratio between the prescribed dose and the defined daily dose and summed the loads over all drugs taken on that day, separately for antiepileptic and psychoactive drugs.

We evaluated, for each patient, whether she or he took antiepileptic or psychoactive medication on the first day in order to determine the overall effect of taking or not taking drugs that are active on the central nervous system in a between-subject design.

In addition, we aimed for a within-subject examination of antiepileptic drug effects by identifying, for each patient, the days with the highest and lowest antiepileptic drug loads.

We identified drugs with a half-life longer than 24 h that were taken by the patients that entered the final analysis (Carbamazepin, Citalopram, Fluoxetin, Perampanel, Sertralin, and Zonisamid). We ensured that only patients were included where the difference in days between the two test sessions that were selected was longer than the half-life and/or where the drug tapering and therefore reduction in drug load was done by changing a co-medication with a shorter half-life.

### 2.7. Seizures

In the epilepsy monitoring unit, the routine procedure includes 24-h recording of video-EEG, using a Micromed S.p.A. (Mogliano: Italy) system, namely a SystemPlus Evolution and an SD LTM 64 Express Amplifier. Twenty-nine electrodes were placed according to the international 10–20 system; Fpz served as ground, and Oz as reference. Impedances were kept below 10 kΩ. The signal was digitally recorded at a 1,024-Hz sampling rate and filtered by 0.1-Hz high-pass and 50-Hz notch. Differential electrocardiogram, electromyogram, and electrooculogram signals were recorded. Patients are monitored by trained staff 24 h a day, providing ictal testing for each seizure or seizure-like event and setting online markers in the EEG recordings. The technical staff at the epilepsy monitoring unit routinely screens the 24-h EEG offline and marks segments with clinical, subclinical, and functional seizures or suspicious events for detailed examination by the neurologist.

An EEG-technician evaluated each of the marked segments (EEG and video) in detail. For each segment, the technician evaluated whether it was an epileptic seizure. Time of seizure onset was compared to the retention interval in order to determine whether there was a seizure in the respective retention interval between learning and recall.

### 2.8. Statistics

The patients' demographic and clinical characteristics were summarized using descriptive statistical methods. Analyses were performed on the raw scores for WHAT, DETAILS, WHEN, ALLOCENTRIC WHERE, and EGOCENTRIC WHERE.

All statistics were conducted with non-parametric statistics in R, since the scale of responses is discrete (i.e., number of remembered items). We used the non-parametric test for multivariate data, npmv (35), with the five subscales as the multivariate response variables. For *post-hoc* characterization of interactions between repeated-measure factors, we used the R-package nparLD for non-parametric analysis of longitudinal data in factorial experiments (36). For interactions with grouping variables, we used a semi-parametric repeated measures ANOVA-type statistic (37). This method is implemented in the R-package MANOVA.RM (38). For correlational analyses, we used Pearson or Spearman correlation, and for comparison of paired samples, the Wilcoxon test.

## 3. Results

### 3.1. Sample

A total of 106 patients had been recruited and participated in the study. The first nine patients participated in a pilot version of the task, such that their data is not comparable with the whole sample. Patients 10 to 24 performed only the first three towns, whereas, for the other 82 patients, all six towns were available. For the purpose of this study, we aimed to use the data maximally and therefore used the full sample of 97 patients and analyzed subsamples with complete data for specific research questions. Clinical details of the whole sample are shown in Table 1, information regarding education and circadian type are given in Table 2, and information about medication in Table 3. A detailed list of the drugs and the number of patients taking each type of drug is given in Table 4.

**Table 1:** Sample: Clinical information.

	**Value**	**Dispersion**
**Mood, 3 missing data**		
Beck Depression Inventory (BDI) score, mean (SD)	10.35	(8.73)
At least minimal depression (BDI > 8), no. (%)	47	(48.45%)
**Diagnosis**		
Focal epilepsy, no. (%)	61	(64.21%)
Generalized epilepsy, no. (%)	13	(13.68%)
No epilepsy, no. (%)	12	(12.63%)
Unclear diagnosis, no. (%)	9	(9.47%)
**Seizures**		
Age at first event, median [range]	19	[0–62]
Years since onset, median [range]	6.5	[0–59]
At least one status epilepticus during life, no. (%)	14	(14.43%)
Status epilepticus within last year, no. (%)	7	(7.22%)
1 seizure during stay, no. (%)	13	(13.40%)
>1 seizure during stay, no. (%)	27	(27.84%)
≥1 night sleep deprivation during stay, no. (%)	30	(30.93%)

**Table 2:** Education and chronotype of participants.

	**Value**	**Dispersion**
**Education, 14 missing data**		
Less than school leaving examination, no. (%)	57	(58.76%)
School leaving examination, no. (%)	21	(21.65%)
University degree, no. (%)	5	(5.15%)
**Chronotype (DMEQ), 1 missing data**		
Clearly morning type, no. (%)	5	(5.15%)
Rather morning type, no. (%)	17	(17.53%)
Neutral type, no. (%)	57	(58.56%)
Rather evening type, no. (%)	13	(13.40%)
Clearly evening type, no. (%)	4	(4.12%)

**Table 3:** Sample: Medication day 1.

	**Value**	**Dispersion**
No antiepileptic drugs, no. (%)	22	(22.68%)
1 antiepileptic drug, no. (%)	41	(42.27%)
2 antiepileptic drugs, no. (%)	22	(22.68%)
3 antiepileptic drugs, no. (%)	11	(11.34%)
Antiepileptic drug tapering during stay, no. (%)	67	(63.81%)
Antiepileptic drug change/uptitration, no. (%)	11	(10.48%)
No psychiatric drugs, no. (%)	79	(81.44%)
1 psychiatric drug, no. (%)	9	(9.28%)
2 psychiatric drugs, no. (%)	4	(4.12%)
3 psychiatric drugs, no. (%)	3	(3.09%)
3 psychiatric drugs, no. (%)	1	(1.03%)

*1 missing data for one patient without epilepsy*.

**Table 4:** Drugs taken by participants on first day of assessment.

**Active compound**	**No. patients**
Amisulpride	1
Aripiprazol	2
Brivaracetam	1
Carbamazepine	11
Clobazam	5
Clonazepam	2
Clozapine	1
Duloxetin	1
Escitalopram	2
Eslicarbazepinacetat	2
Ethosuximide	1
Fluoxetine	1
Hydromorphone	2
Lacosamide	12
Lamotrigine	13
Levetiracetam	51
Lorazepam	1
Mirtazepine	2
Nitrazepam	2
Oxcarbazepine	9
Perampanel	3
Pregabalin	4
Quetiapin	2
Risperidone	3
Rufinamid	1
Sertralin	5
Topiramate	3
Trazodone	3
Triazolam	1
Valproic acid	9
Venlafaxine	4
Zolpidem	1
Zonisamid	6

### 3.2. Test Sessions

Not all patients participated in all six test sessions. The first 15 patients included in this sample were tested only with the first three versions. In several cases, the stay in the epilepsy monitoring unit was shortened by one day, e.g., when Monday was a holiday. Furthermore, since participation was voluntary, patients occasionally decided not to take part in single sessions. When a patient had visitors at the time of examination, we tried to do the tests later, but, if impossible, the session was missed. When there was a seizure shortly before the scheduled examination, patients sometimes refused to take part because of feeling tired or uncomfortable after seizures. Finally, several patients stopped participating at all after the first sessions.

Table 5 shows the number of patients that participated in each session. The number of patients declined with the number of towns, that is, over sessions, as patients were more likely to drop out later on.

**Table 5:** Number of patients per test session.

**Town**	**Immediate**	**Delayed**
1	95	90
2	87	79
3	85	81
4	66	61
5	60	57
6	50	45

### 3.3. Confounding Variables

#### 3.3.1. Habituation Over Test Sessions

In order to determine the potential bias from unspecific learning effects due to habituation over the course of the six-test session, we extracted *N* = 20 patients without a change in medication, out of which *N* = 9 had a complete dataset (i.e., participated in all 6 towns). All but one of these patients took no drugs at all; the exception had a constant antiepileptic drug load which corresponded to a defined daily dose of 2. Table 6 shows the results of the non-parametric test nparLD for changes over the six test sessions in this sample. Though the results were significant at *p* < 0.05 (uncorrected) for the subscales WHAT, DETAILS, and EGOCENTRIC WHEN, these scales showed a decrease rather than an increase in performance over the six test sessions, with the highest performance in test session 1. Thus, in the absence of a drug effect, there is no overall learning effect due to unspecific factors across the six parallel versions of the VR task.

**Table 6:** Control group effects of habituation over the week.

**Town**	**What**	**Details**	**When**	**Ego**	**Allo**
*F*-value	3.53	3.00	0.71	2.77	2.27
*p*-value	0.02	0.03	0.57	0.03	0.09
Rank means					
1	32.83	32.89	29.67	33.33	24.94
2	29.22	31.78	27.83	29.44	29.39
3	31.00	30.39	22.78	24.39	30.50
4	19.89	15.33	24.83	20.11	23.67
5	31.72	28.61	33.83	31.39	35.89
6	20.33	26.00	26.06	26.33	20.61

*Ego, egocentric where; Allo, allocentric where*.

#### 3.3.2. Time of the day and Chronotype

The type of retention interval can be night or day, depending on whether delayed recall occurs in the evening or morning. A significant interaction between time of day and retention would indicate a significant difference between daytime and nighttime retention. Chronotype is considered an important moderator, especially in regard to the morning session, which was scheduled at 7:00 a.m. We calculated one non-parametric multivariate analysis of the repeated measure factor time of day (morning vs. evening) and one for chronotype (morning, neutral, evening), each in interaction with retention. For this purpose, we restricted the dataset to two towns in order to have each of one night and one day as the retention interval. We used towns 1 and 2 to examine the effects of time of the day and chronotype because these towns show a relative advantage over other towns in terms of complete datasets. This resulted in a sample of *N* = 59.

The multivariate analysis of variance for towns 1 and 2 yielded no significant effect of time of day [*F*_(3.22, 753.55)_ = 0.19; *p* = 0.972] and no significant effect of chronotype [*F*_(8.65, 607.94)_ = 0.60; *p* = 0.767]. Since a potential interaction between chronotype or time of day would severely bias the results, we performed subscale-wise non-parametric analysis of variance (MANOVA.RM). Table 7 shows the analysis of the interaction between time of day and chronotype, indicating no significant effects of interactions.

**Table 7:** Non-parametric analysis of factor daytime in interaction with chronotype.

	**Daytime**		**Chronotype**		**Interaction**	
**Subscale**	**ATS**	***p***	**ATS**	***p***	**ATS**	***p***
What	0.40	0.526	0.53	0.556	0.912	0.395
Details	0.25	0.618	0.97	0.369	0.25	0.709
When	1.22	0.270	2.54	0.100	0.32	0.667
Egocentric	1.67	0.196	0.16	0.829	0.34	0.944
Allocentric	0.28	0.594	0.06	0.918	0.70	0.457

*ATS, ANOVA-type test value; p, p-value; df: (1,1)*.

*Bonferroni-corrected critical alpha level is p < 0.01*.

#### 3.3.3. Age and Gender

In order to determine group-level effects of age and gender, we selected the town with the most responses, that is, town 1 with 90 responses for both sessions (immediate and delayed recall). Additionally, town 1 is not biased by the effects of sleep deprivation and tapering/change of medication. We divided all participants into two groups by the median age (27.5). There was no significant effect of gender, nor was there an interaction between the factors gender and age [*F*_(3.25, 564.87)_ = 0.15; *p* = 0.990]. We found a significant effect of age group [*F*_(3.55, 624.46)_ = 9.37; *p* < 0.001]. The *post-hoc* Spearman correlations for subscales with age are shown in Table 8. Recall on the subscale DETAILS correlated significantly during immediate and delayed recall with age, as well as delayed recall on the subscales WHAT and ALLOCENTRIC. The negative correlation coefficients indicate that older individuals recall fewer items.

**Table 8:** Correlation with age.

	**Immediate**		**Delayed**	
**Subscale**	**rho**	***p***	**rho**	***p***
What	–0.29	0.017	–0.36	0.001
Details	–0.40	<0.001	–0.36	0.001
When	–0.18	0.213	–0.16	0.165
Egocentric	–0.25	0.037	–0.20	0.071
Allocentric	–0.19	0.132	–0.33	0.003

*Bonferroni-corrected alpha level is p < 0.005*.

#### 3.3.4. Depression

In order to determine the effect of depression, we again selected town 1. There was no significant effect of depression when dividing patients into the five groups according to the BDI manual of no, mild, minimal, moderate, and severe depression [*F*_(19.31, 334.11)_ = 1.74; *p* = 0.087]. The tendency was such that patients with moderate to severe depression performed worse than patients with no or only mild depression.

### 3.4. Effect of Epilepsy

In order to detect the group effects of epilepsy, town 1 with 90 responses was used. After excluding participants with missing data on delayed recall and participants where the diagnosis was unclear, 84 participants remained for analysis. Among them, 11 were used as a control group without epilepsy. In this group, six were diagnosed with “no epilepsy,” three were diagnosed with functional events, e.g., in situations of high workload, and two were released with no diagnosis because, though they had a single event that was possibly a seizure, no additional abnormalities were found during monitoring.

For the purpose of determining the effects of epilepsy, we performed four analyses. Therefore, we corrected for multiple comparisons, obtaining a Bonferroni-corrected critical threshold of α < 0.0125. In order to assess a general effect of epilepsy, we analyzed data from the town with the most responses, that is, town 1. We examined the effect of epilepsy by applying a multivariate non-parametric analysis of variance to immediate and delayed recall of town 1, with retention as a repeated-measures factor. We performed this analysis for four different grouping factors. First, we used the diagnosis of epilepsy vs. no epilepsy as a group factor. Second, we compared patients with temporal lobe epilepsy, grouped by lateralization (left, right), to other patients with epilepsy. Third, we grouped patients with epilepsy by median-split into two groups according to age of onset, and fourth, according to years since onset.

There was no effect of epilepsy when comparing patients with and without epilepsy on all subscales in interaction with retention [*F*_(2.89, 211.97)_ = 0.51; *p* = 0.735]. We performed a subanalysis where we extracted patients with temporal lobe epilepsy with focus on the left side (*N* = 14) and focus on the right side (*N* = 12) and compared them to the control group without epilepsy (*N* = 11). The multivariate analysis of variance for all subscales and retention as a repeated-measures factor revealed no significant effect for temporal lobe epilepsy groups [*F*_(6.08, 213.63)_ = 0.49; *p* = 0.839].

In order to determine the effect of age at onset as well as the years since onset of epilepsy, we divided the group into two groups by the median of age at onset (19 years) and by the median number of years since onset (6 years). We excluded patients without epilepsy and where this information was unavailable, resulting in a sample of *N* = 84. The multivariate analysis of variance for all subscales and retention as a repeated-measures factor revealed no effect of age at onset [*F*_(3.29, 532.82)_ = 0.58; *p* = 0.670] and no effect of years since onset [*F*_(3.36, 551.15)_ = 1.32; *p* = 0.226].

### 3.5. Effect of Medication

We examined the effect of medication with two analyses, resulting in a Bonferroni-corrected critical threshold of α < 0.025. In order to determine a general effect of drugs, we divided all participants into those who did not take any drugs that are active on the central nervous system and those who took at least one such drug. We compared these two groups alongside the factor retention (immediate vs. delayed) in a multivariate analysis of variance for the five subscales of the virtual reality test.

Some patients took psychoactive drugs where the use is not always clear, e.g., Benzodiazepines may be used for the management of epilepsy or for psychiatric purposes. Therefore, we compared performance between patients taking no drugs (*N* = 19) and patients taking antiepileptic and/or psychoactive drugs (*N* = 69). We excluded one patient where drug information was missing and one patient who was an outlier due to taking seven drugs (antiepileptic drugs and psychoactive drugs combined). The average drug load was 0.22 for psychiatric drugs (SD = 0.62) and 1.17 for antiepileptic drugs (SD = 1.27). There was a significant overall effect of medication on task performance across all subscales of the virtual reality task [*F*_(2.84, 330.43)_ = 5.26; *p* = 0.005].

Since this effect could be mixed up with the effect of psychoactive drugs or with the severity of epilepsy, we examined dosage change effects within subjects for antiepileptic drugs only. Psychoactive drugs were not changed throughout the week, while antiepileptic medication was changed. We found pairs of sessions with no missing data and where drug load, calculated as the proportion of the prescribed drug in the defined daily dose, summed over all antiepileptic drugs, differed between the session with maximum and minimum drug load. In some cases, there were more days with a similar maximum drug load. In that case, we took the latest day throughout the week, in order to avoid a systematic effect of task version, since in most patients, the maximum drug load was administered on the first day of testing. Similarly, we strived to find the first day with minimum drug load in order to avoid an effect of habituation, leading to systematically improved scores in low-drug load test sessions.

**Figure 4:**
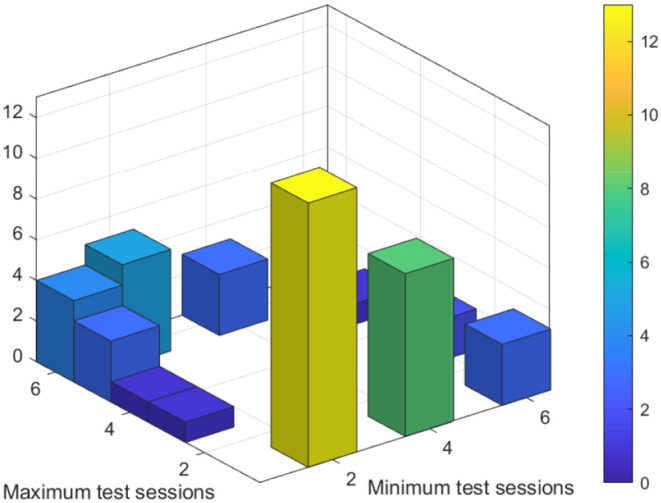
Test sessions with minimum and maximum antiepileptic drug load.

There were 62 patients with at least two complete sessions of immediate and delayed recall and information on drug load, and 42 patients were found with a real difference in antiepileptic drug load. A real difference means that we selected only those patients where the halflife of the drugs with dosage changes was smaller than the time difference between the two sessions of the memory tests. Figure 4 shows the distribution of selected test sessions for minimum and maximum drug load. Most patients had the maximum drug load on the first session and the minimum drug load on the next day in session 2 (*N* = 13) or in session 4 (*N* = 8).

**Figure 5:**
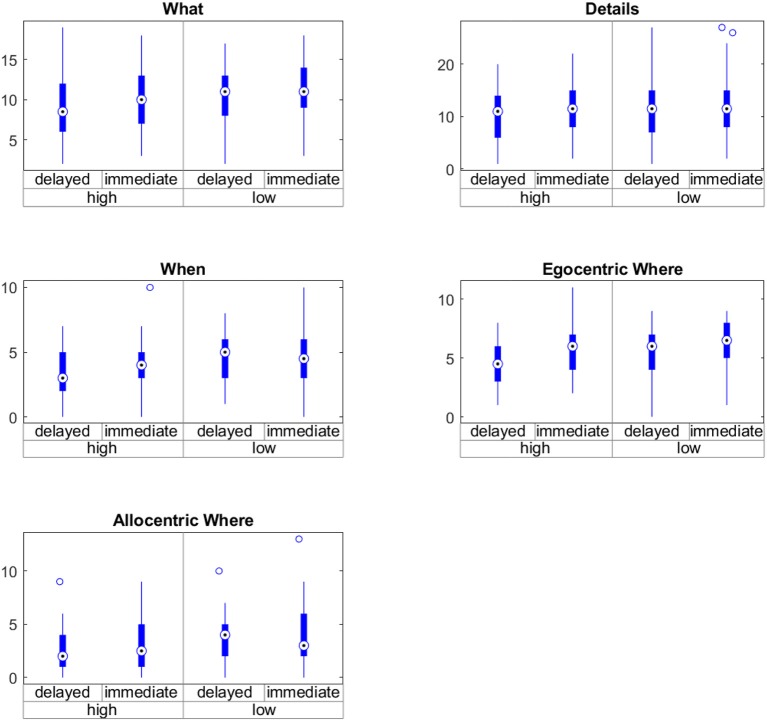
Interaction between time of testing (immediate vs. delayed) and drug load (high vs. low) on the subscales of the virtual town episodic memory test.

The average minimum drug load was 0.32 (SD = 0.64), and the average maximum drug load was 1.39 (SD = 1.25). The average difference between the minimum and maximum drug load was 1.06 (SD = 0.91); the minimum difference was 0.23, and the maximum 3.9. There was a significant effect of medication on task performance [*F*_(2.56, 424.38)_ = 4.73; *p* = 0.01]. The results of the non-parametric repeated-measures ANOVA for the factors medication and retention for all subscales are shown in Table 9 and Figure 5. In contrast, there was no significant difference in learning-time, i.e., the time spent on navigating through the virtual town, between sessions with low and high drug load (Wilcoxon signed-rank test W = 343, *p* = 0.26).

**Table 9:** Effect of drug load.

	**Medication**		**Retention**		**Interaction**	
**Subscale**	**ATS**	***p***	**ATS**	***p***	**ATS**	***p***
What	7.43	0.006	22.27	<0.001	1.16	0.282
Details	1.42	0.234	8.03	0.005	0.26	0.611
When	7.90	0.005	5.86	0.015	1.73	0.189
Egocentric	5.39	0.020	24.57	<0.001	1.66	0.198
Allocentric	12.23	<0.001	1.44	0.230	0.30	0.582

*ATS, ANOVA-type statistic; Bonferroni corrected p < 0.01*.

**Figure 6:**
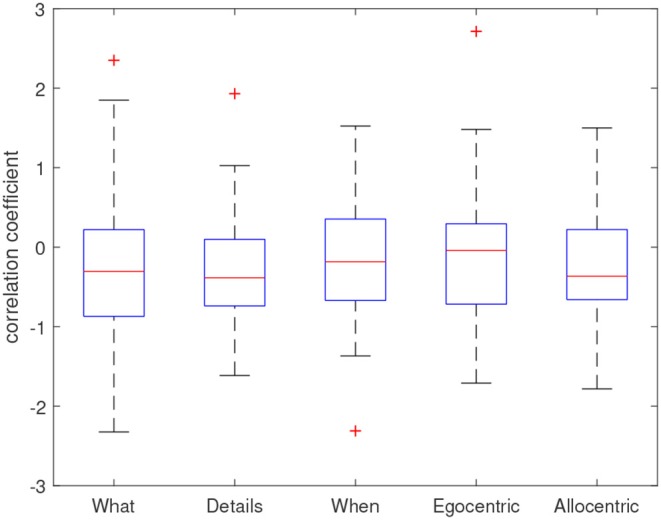
Distribution of correlation coefficients across patients. Coefficients represent Fisher-r-to-z-transformed correlation coefficients resulting from a patient-wise Pearson correlation between antiepileptic drug load and memory scores over all available test sessions.

In order to examine the relation between change of antiepileptic drug load and change in memory scores, we performed patient-wise correlation between daily determined drug load and daily obtained memory performance on the five subscales for patients with at least three immediate test sessions and a change in drug load (*N* = 56). The resulting correlation coefficients of these patient-wise correlations, after Fisher-r-to-z-transformation, are presented as boxplots in Figure 6 and demonstrate a tendency toward a negative relationship between drug load and memory performance.

Since the sample took a wide variety of different antiepileptic drugs, we conducted a few tests to check whether the test result would be different if we (1) examined the sample without the three patients who took Topiramate, a drug which is well-known to affect cognition (9) and (2) by examining only patients who only took Levetiracetam or Lamotrigin, both drugs that have been reported to have no cognitive side effects (9). It was not possible to perform further subanalyses for specific drugs, as out of the 20 patients who took only one drug, 12 took Levetiracetam, five took Lamotrigin, two took Oxcarbamazepin, and one took Pregabalin. Thus, the sample size was not sufficient for the other drugs.

The effect was still significant when excluding the three patients on Topiramate [*F*_(2.49, 383.26)_ = 5.1; *p* = 0.006]. However, performing the same test on the sample that only took Levetiracetam [*F*_(2.56, 110.08)_ = 1.57; *p* = 0.179] or Lamotrigin [*F*_(2, 33.97)_ = 0.34; *p* = 0.813] yielded no significant effect.

### 3.6. Effect of Seizures and Lesions

In order to control for the effect of seizures, we repeated the non-parametric test for multivariate data examining the effect of drug load in interaction with retention but extended it with a factor that indicates whether there was an epileptic seizure in the retention interval between learning and recall. The result was strikingly similar [*F*_(2.76, 457.76)_ = 4.56; *p* = 0.006]. Indeed, there were only three intervals with seizures in the high-drug load condition and four intervals with seizures in the low-drug load condition. All intervals included at maximum one seizure, such that examination of the effect of a higher number of seizures was not necessary. Excluding these intervals left the effect unchanged [*F*_(2.56, 424.38)_ = 4.73; *p* = 0.009].

In order to control for the effect of structural abnormalities, we repeated the non-parametric test for multivariate data examining the effect of drug load in interaction with retention but extended it with a factor that indicates whether structural imaging with MRI has revealed a lesion. Out of the 42 patients, 10 were diagnosed with abnormalities in the MRI, 13 with non-lesional MRI, and, for the others, no MRI was conducted. We included them as a separate group, with the grouping factor lesion (lesional, non-lesional, unknown). Again, the inclusion of the factor lesion did not change the result [*F*_(3.21, 533.59)_ = 4.09; *p* = 0.008].

## 4. Discussion

In this study, we provided direct evidence that antiepileptic drug load negatively influences episodic memory in patients with epilepsy. Episodic memory performance was not determined by any other factor but age, especially not by epilepsy. Even when assessing the subsamples of patients with temporal lobe epilepsy, there was no effect of lateralization. In contrast, there was a significant effect of medication.

In patients with epilepsy, many factors may have a negative influence on memory performance (39). There is evidence for the negative influence of pathological changes in the brain (40), genetic background (41, 42), age at onset (43), duration of epilepsy (44, 45), epilepsy-specific disturbances of sleep (46), psychosocial consequences of the disorder, including lower levels of education and poor social integration (47, 48), and seizures (6). Our results suggest that the effect of drugs should be taken into account when examining the influence of other factors.

### 4.1. Structural Damage or Drugs?

Patients with temporal lobe epilepsy suffer from long-term memory problems and especially from poor episodic memory (49). Previous research demonstrated that patients with temporal lobe damage on the right side perform significantly worse than patients with damage on the left temporal lobe in topographical tasks (23). Weniger and Irle (18) provided evidence that in addition to hippocampal mechanisms, the somatosensory cortex may play a role in the impaired spatial memory of patients with temporal lobe epilepsy. The authors reported a relation between smaller volumes in the left-ided post-central gyrus and worse performance on a virtual maze test.

In contrast, Cánovas et al. (21) reported no effect of lateralization on performance. The detection of lateralization effects seems to depend on detailed information about the localization and nature of damage. Butler et al. (50) found no gross anatomical correlate of epilepsy-related long-term amnesia and hypothesized that subtle changes could be the cause for it. Although the role of the hippocampus is an established fact in science (17), we could speculate that a reduced number of episodic memories in patients with epilepsy may be additionally affected by medication. Benzodiazepine drugs, especially, are known to cause episodic memory impairment (51) combined with impairment of visual perception (52). Furthermore, benzodiazepines can induce amnestic cognitive impairments, with evidence of a dose-response relationship (53). Benzodiazepines are used in the management of epilepsy (54) and especially for the treatment of convulsive status epilepticus in children and adults (55). Gascoigne et al. (56) reported autobiographic episodic memory being impaired in children with temporal lobe epilepsy compared to controls. Healthy control children demonstrate the development of memory by an increasing number of episodic details recalled from 6 to 16 years of age. Children with temporal lobe epilepsy are impaired in this type of development, but the authors did not find relationships between richness of episodic recall and epilepsy factors, including structural hippocampal abnormalities. Once again, we can assume that children with epilepsy are treated by medication. In turn, we can speculate that antiepileptic medication impairs the development of memory, as it is known that several antiepileptic drugs have at least short-term effects on memory in children (57). Future long-term studies are needed to differentiate the effect of medication from the effect of epilepsy during critical periods of brain development in childhood.

In a study by Voltzenlogel et al. (58), autobiographic episodic memory was impaired in drug-resistant patients with temporal lobe epilepsy compared to drug-responsive patients. The average difference in numbers of drugs per patient between the two groups was 1.15. According to our data, the difference in medication should be taken into account when examining cognitive performance.

Correlation of memory impairment with the duration of epilepsy and age at onset of epilepsy has led to contradictory findings in the past (39). We could not replicate a relation between episodic memory impairment and duration of epilepsy or age at onset. The effect of duration of epilepsy may interact with the frequency of seizures (39), and we speculate that drug load also plays a role.

In our study, we grossly controlled for the effect of temporal lobe epilepsy and the effect of lesions. TLE did not affect memory, and a lesional MRI did not interact with the effect of drug load. It is likely that the heterogeneity of lesions, even in the TLE group, explains our missing the effect. A larger sample size would be needed to group patients according to specific lesions and their impact and potential interactions with drugs. The recently published, largest neuroimaging study of epilepsy to date provided evidence for structural abnormalities on the common epilepsies (59). Specifically, this study also identified associations between duration of epilepsy and several affected brain regions. It is possible that the structural change interacts with the intake of drugs, a hypothesis that could be addressed in future large-scale studies.

There are only a few studies investigating the adverse effects of antiseizure drugs in adults with refractory epilepsy and their relationship with AED load. In a large, multicentric study, Canevini et al. (60) could not detect a relationship between adverse effects and co-prescribed drugs or drug load. As the adverse events profile that was used in this study is very general, it would be highly warranted to differentiate the effects in specific domains for this patient population in more detail.

### 4.2. Subscales of Episodic Memory

The test based on virtual towns in our study was inspired by a former study in patients with mild cognitive impairment and Alzheimer's disease (19). The advantage of this test is that episodic memory can be separately examined on subscales of remembered semantic contents (WHAT, DETAILS), information in time (WHEN), and space (WHERE). A closer look at the data in a within-subject design revealed that antiepileptic drugs differentially affected spatial aspects of episodic memory, with a stronger effect on allocentric spatial information. Further significant effects were found for elements seen in the environment (WHAT) and timing of events (WHEN). It should be noted, however, that egocentric memory was tested with a question that allows guessing, which is likely to generally downgrade the accuracy of measurement of memory performance on this scale.

Spatial episodic memory has been intensively examined, and it is established knowledge that spatial learning is dependent on hippocampal place cells (17). Prior research in patients with temporal lobe epilepsy used two different tasks, a virtual park and a virtual maze, in patients with temporal lobe epilepsy (22). The authors demonstrated a dissociation between egocentric and allocentric information, which seem to be differentially affected by specific lesions of the parahippocampal gyrus. Both of these tasks rely on the ability to navigate through the virtual environment, which depends on age and experience with computerized virtual environments such as games. Especially if the time needed to navigate to an object is used as a measure, the familiarity of the patient with usage of the joystick or the cursor keyboard can cause significant bias. An alternative is to ask the patient about his/her experience. Cánovas et al. (21) and Rosas et al. (24) asked participants about prior experience and measured travel distance and errors. The task used in the presented study is advantageous, as navigation performance does not directly affect any measure of memory. In addition, the time spent in the learning environment was no different between sessions with high and low drug load.

### 4.3. Failure of Encoding or Failure of Recall?

Delayed recall was subject to forgetting, but forgetting did not interact with medication. This suggests that, actually, medication does not accelerate forgetting but deteriorates either encoding, i.e., interferes with the learning of new contents, or recall, i.e., interferes with access to learned contents. Episodic memory relies on a wider focus of attention than semantic memory, where single items have to be learned one by one. In our virtual reality town, participants have to monitor multiple events and details at the same time, which requires both attention and rapid encoding of multiple contents that are fed in parallel to the stream of information. We could speculate that the effect of medication narrows down the ability to monitor and memorize multiple contents at the same time. Prior research revealing attention deficits due to antiepileptic medication supports this interpretation (9). On the other hand, learning might be intact, but recall in general could be affected. We suggest that neurophysiological studies should determine whether the effect is due to altered brain activity during learning or recall.

### 4.4. Limitations and Future Studies

In our study, we examined a heterogeneous group of patients with epilepsy, and we did not differentiate all subgroups of active agents in the drugs because of a lack of large sample sizes for most individual drugs or drug combinations. There is evidence that the type of drug determines whether memory is affected, and if so, whether it is deterioriated or enhanced (9). In our sample, the main effect of drug load was not only due to the well-known negative effect of Topiramate. However, when examining only those patients who are on treatment with Levetiracetam or Lamotrigin, there was no significant effect of drug load. While this result is highly plausible and well in line with the literature (9), it is not recommendable to over-interpret this result, as the power was considerably lower for these sub-samples of patients. In general, we replicated the effect of drug load, according to previous research suggesting that medication is a major determinant for memory impairment (10). In order to differentiate the subtle effects and interactions of active compounds in drugs, a sample at the range of several hundreds of patients would be needed [such as, for example, >800 patients in the large study by Witt et al. (10)]. With the present protocol of intensive examination, twice a day, that was not part of the clinical routine, we approached the limits of feasibility. Future multicentric studies that incorporate the virtual reality test for episodic memory into the everyday testing routine could allow a sufficiently large sample to be achieved for the examination of specific combinations of drugs.

Statistical power is an important factor to consider when interpreting our results. Although we did not provide a formal power analysis, the negative results must be considered in relation to the varying sample sizes for the various research questions. Especially, the analysis of subsamples of patients with temporal lobe epilepsy and patients taking specific drugs must be handled with the limitation of smaller sample size and, therefore, lower power.

We controlled for the effect of seizures, as seizures are known to interfere with memory (6). Drug tapering is conducted in order to provoke seizures, which means that on days with low drug load, performance could be even lower than usual because of seizures. In our data, the overall number of intervals including seizures was very small, and we found no effect of seizures, nor did the result change when excluding all intervals including seizures. Since the number of seizures was so low in the presented sample, we cannot conclude that seizures do not have an effect on memory, because the data is not sufficiently powered for this type of analysis.

Ideally, we would have determined test-retest reliability for the task, which was not possible as we did not have retest sessions for the same towns. Furthermore, the results of the small control group for the towns indicate that the six versions are not parallel. However, the effect of lower performance toward the end of the week excludes a habituation effect. A habituation effect could mimic our results, as, in most patients, the assessment with lower drug load is later in the week. However, a worsening in performance counteracts the effect we reported. Our result cannot be due to the systematic decline over the week that was observable in the control group and thus in the absence of drug changes or seizures. It is highly warranted to validate the six towns in terms of test-retest reliability and to improve parallelism in future studies.

Our data did not reveal an interaction between retention and medication. We examined a 12-h retention interval, which is longer than intervals used in classical neuropsychological examination settings. Still, 12 h might be too short to detect the effects of epilepsy, which happen at long-term delays at the range of several days for non-visual material (39).

## 5. Conclusions

This study provides direct evidence for episodic memory impairment from antiepileptic drugs. The presented approach of repeated examination in the epilepsy monitoring unit is a feasible design to obtain within-patient data in studies that examine the cognitive effects of drugs. Patients' concerns about memory impairment influence treatment preference (61). The demand from patients for more emphasis on quality increases the pressure to exactly determine the minimum effective dose. As this study is limited in sample size, future studies should systematically gather samples of patients with specific drugs and combinations of drugs in order to differentiate drugs with less harmful neurocognitive profiles.

## Data Availability Statement

The raw data supporting the conclusions of this article will be made available by the authors, without undue reservation, to any qualified researcher.

## Ethics Statement

The studies involving human participants were reviewed and approved by ethical committee Salzburg (415- E/1755/24-2018). The patients/participants provided their written informed consent to participate in this study.

## Author Contributions

YH: conceptualization, methodology, formal analysis, investigation, writing–original draft, visualization, project administration, and funding acquisition. CH: validation, investigation, data curation, writing–review and editing, and project administration. FS: software, validation, investigation, data curation, visualization, and writing–review and editing. GP: conceptualization, methodology, and writing–review and editing. ET: conceptualization, resources, writing–review and editing, and supervision.

### Conflict of Interest

ET has acted as a paid consultant to Eisai, EVER Neuro Pharma, Biogen Idec, Medtronics, Bial, and UCB and has received speakers' honoraria from Bial, Eisai, Boehringer Ingelheim, Biogen, Newbridge, Novartis, and UCB Pharma in the past 3 years. ET has received research funding from UCB Pharma, Biogen, Novartis, Bayer, Eisai, Red Bull, Merck, the European Union, the Austrian Science Fund (FWF), and Bundesministerium für Wissenschaft und Forschung. ET is also one of the investigators planning the ESET Trial and a member of the Task Force on Classification of Status Epilepticus of the International League Against Epilepsy. The remaining authors declare that the research was conducted in the absence of any commercial or financial relationships that could be construed as a potential conflict of interest.
